# VA residential substance use disorder treatment program providers’ perceptions of facilitators and barriers to performance on pre-admission processes

**DOI:** 10.1186/s13722-017-0075-z

**Published:** 2017-04-04

**Authors:** Laura S. Ellerbe, Luisa Manfredi, Shalini Gupta, Tyler E. Phelps, Thomas R. Bowe, Anna D. Rubinsky, Jennifer L. Burden, Alex H. S. Harris

**Affiliations:** 1 Center for Innovation to Implementation, Department of Veterans Affairs (VA) Palo Alto Health Care System, 795 Willow Road (MPD-152), Menlo Park, CA 94025 USA; 2 Department of Medicine, University of California, San Francisco and the San Francisco VA Medical Center, San Francisco, CA USA; 3 Salem VA Medical Center, Salem, VA USA

**Keywords:** Substance use disorders, Residential treatment, Standards of care, Quality measurement, Quality improvement

## Abstract

**Background:**

In the U.S. Department of Veterans Affairs (VA), residential treatment programs are an important part of the continuum of care for patients with a substance use disorder (SUD). However, a limited number of program-specific measures to identify quality gaps in SUD residential programs exist. This study aimed to: (1) Develop metrics for two pre-admission processes: *Wait Time* and *Engagement While Waiting*, and (2) Interview program management and staff about program structures and processes that may contribute to performance on these metrics. The first aim sought to supplement the VA’s existing facility-level performance metrics with SUD program-level metrics in order to identify high-value targets for quality improvement. The second aim recognized that not all key processes are reflected in the administrative data, and even when they are, new insight may be gained from viewing these data in the context of day-to-day clinical practice.

**Methods:**

VA administrative data from fiscal year 2012 were used to calculate pre-admission metrics for 97 programs (63 SUD Residential Rehabilitation Treatment Programs (SUD RRTPs); 34 Mental Health Residential Rehabilitation Treatment Programs (MH RRTPs) with a SUD track). Interviews were then conducted with management and front-line staff to learn what factors may have contributed to high or low performance, relative to the national average for their program type. We hypothesized that speaking directly to residential program staff may reveal innovative practices, areas for improvement, and factors that may explain system-wide variability in performance.

**Results:**

Average wait time for admission was 16 days (SUD RRTPs: 17 days; MH RRTPs with a SUD track: 11 days), with 60% of Veterans waiting longer than 7 days. For these Veterans, engagement while waiting occurred in an average of 54% of the waiting weeks (range 3–100% across programs). Fifty-nine interviews representing 44 programs revealed factors perceived to potentially impact performance in these domains. Efficient screening processes, effective patient flow, and available beds were perceived to facilitate shorter wait times, while lack of beds, poor staffing levels, and lengths of stay of existing patients were thought to lengthen wait times. Accessible outpatient services, strong patient outreach, and strong encouragement of pre-admission outpatient treatment emerged as facilitators of engagement while waiting; poor staffing levels, socioeconomic barriers, and low patient motivation were viewed as barriers.

**Conclusions:**

Metrics for pre-admission processes can be helpful for monitoring residential SUD treatment programs. Interviewing program management and staff about drivers of performance metrics can play a complementary role by identifying innovative and other strong practices, as well as high-value targets for quality improvement. Key facilitators of high-performing facilities may offer programs with lower performance useful strategies to improve specific pre-admission processes.

## Background

In the United States Department of Veterans Affairs (VA), residential rehabilitation treatment programs are an integral part of the continuum of care for patients with a mental health or substance use disorder (SUD) [1]. Research on the effectiveness of residential treatment for substance use disorders is mixed but overall suggests that it fills a particular niche and is a valuable option for some types of patients [2]. Veterans with SUD who need specialized, 24/7 structure and support may seek treatment in one of the VA’s 63 SUD Residential Rehabilitation Treatment Programs (SUD RRTPs) or 34 Mental Health Residential Rehabilitation Treatment Programs (MH RRTPs) with a SUD track. Each of the 97 programs offers recovery-oriented, patient-centered care, including evidence-based individual, and group psychotherapy and pharmacotherapy, to address SUD, co-occurring mental health conditions and other severe psychosocial concerns such as homelessness and unemployment.

Despite their place in the continuum of care, residential programs are resource-intensive and costly, with associated health care costs (including indirect costs) estimated to be roughly $210 million for VA in FY 2014, posing a fiscal challenge for a capitated healthcare system with competing demands [3]. Patient demand for SUD RRTPs, in particular, has increased steadily, with a 10.7% increase in admissions from FY 2012 to FY 2014 [4].

In order to reduce undesirable variability in quality of care across VA residential SUD treatment programs, core standards and practices for these programs have been codified in two VA handbooks, the Uniform Mental Health Services Handbook (revised in 2008) and the MH RRTP Handbook (published in 2010) [1, 5]. These handbooks outline requirements to ensure that Veterans have access to comprehensive, evidence-based SUD and other mental health services. For example, all residential programs are required to provide or arrange pre-admission treatment and case management to Veterans waiting for admission [1, 5].

VA Mental Health Services and the VA Office of Mental Health Operations have made adhering to these standard requirements for care delivery, managing costs, and improving outcomes their highest priority. Recognizing the need for a mechanism to routinely monitor the performance of all mental health services, including those provided in residential treatment programs, the Office of Mental Health Operations developed the Mental Health Information System (MHIS) [6]. The MHIS is an informatics dashboard comprised of dozens of metrics, including 15 metrics assessing access to and the quality of SUD treatment. However, only two metrics focus specifically on *residential* SUD treatment: (a) an access measure—the proportion of patients with a SUD diagnosis that receive care in a residential SUD treatment program, and (b) average length of stay in a residential SUD treatment program among patients admitted. While these measures are useful, VA would benefit from a comprehensive suite of measures to monitor other important structures, processes and outcomes of residential SUD treatment programs.

Therefore, we sought to develop metrics using electronic health records data for VA residential SUD treatment programs in three key domains: pre-admission (e.g., wait time, engagement while waiting), in-treatment (e.g., use of addiction pharmacotherapy), and post-discharge (e.g., outpatient SUD follow-up, readmission, subsequent detoxification episodes). After calculating the metrics for all programs, we interviewed residential SUD treatment program management and front-line staff. This enabled us to validate that the metric data corresponded with their day-to-day experience, and to learn their impressions regarding the facilitators and/or challenges that may have impacted their program’s performance on metrics for which the program was substantially higher or lower than the VA national average. In this paper, we focus on the *Pre*-*Admission* domain and the corresponding qualitative data which describe factors that may explain observed variability, in order to learn about potential innovative practices, and identify possible areas for improvement.

## Methods

### Study population

National data collected by the VA Northeast Program Evaluation Center (NEPEC) were used to identify 97 VA residential SUD treatment programs (63 SUD RRTPs; 34 MH RRTPs with a SUD track). Patients receiving treatment from these programs were then located in VA electronic health records data using combinations of station and specialty treatment (bed section) codes.

### Developing and calculating metrics

Quality measures prioritized for metric development were chosen in collaboration with our operational partners in VA Mental Health Services (MHS) and the Office of Mental Health Operations (OMHO). The selected metrics include SUD-specific versions of other Mental Health Information System (MHIS) metrics, and processes of care emphasized in program evaluation reports [e.g., OIG (Department of Veterans Affairs (VA) Office of the Inspector General (OIG)), 2009 and 2011] [7, 8]. These new metrics capture critical aspects of pre-admission processes, in-treatment processes and practices, and post-discharge follow-up.

Two *pre*-*admission* processes are the focus of this paper—wait time (two metrics) and engagement while waiting for admission (three metrics). Descriptions of how these metrics were operationalized are presented in Table 1. If the percent of admissions with a pre-admissions screening visit was less than 25% for a program then wait time and engagement metrics were not calculated since the screening visit (stop code 596) marked the start of the clock for those metrics. Since these wait time and engagement while waiting data metrics rely on the use of the 596 stop code, non-use or low use of the code would render it difficult or impossible to calculate and meaningfully interpret a program’s performance. While 25% is admittedly arbitrary, the threshold was chosen with consultation from our operational partners. For programs without valid metric data, participants were interviewed about factors that impacted their program’s low screening rates, as well as typical wait time and engagement while waiting.

**Table 1:** Pre-admission metric definitions and national averages for SUD RRTPs and MH RRTPs with a SUD track

Metric	Definition	SUD RRTPs*	MH RRTPs with a SUD track*
		Data (SD)	Data (SD)
Wait time			
Mean days from screen to admission (based on patient-level wait times from 42 SUD RRTPs and 19 MH RRTPs with a SUD track)	Time (days) between screen and admission, averaged at the program level, for Veterans screened with a 596 stop code** and admitted to a residential SUD treatment program	17 days (35.46)	11 days (34.49)
Percent of admissions with >7 day wait (for SUD RRTPs, based on 1047 out of 4550 Veterans, and for MH RRTPs with a SUD track, based on 676 out of 1780 Veterans)	Percent of Veterans admitted to a residential SUD treatment program who waited >7 days after screening to enter the program	23%	38%
Engagement while waiting			
Percent of weeks with any outpatient SUD or MH contact (based on patient-level number of waiting weeks)	Among Veterans who waited >7 days between screening and admission to residential SUD treatment, percent of weeks while waiting in which Veterans received at least one outpatient SUD or MH contact	55% (.336)	47% (.389)
Percent of weeks with any outpatient SUD contact only (based on patient-level number of waiting weeks)	Among Veterans who waited >7 days between screening and admission, percent of weeks while waiting in which Veterans received at least one outpatient SUD contact	39% (.355)	24% (.343)
Percent of weeks with any outpatient MH contact only (based on patient-level number of waiting weeks)	Among Veterans who waited >7 days between screening and admission, percent of weeks while waiting in which Veterans received at least one outpatient MH contact	31% (.31)	35% (.34)

Data are from fiscal year (FY) 2012

*SUD* substance use disorder, *RRTP* Residential Rehabilitation Treatment Program, *MH* mental health, *SD* standard deviation

* Includes programs with less than 10 admissions

** The 596 clinic stop code was activated in FY 2009 and indicates RRTP Admission Screening Services. Per the VHA Handbook 1162.02, this code must be used to document a screening for residential treatment. Any outpatient SUD or MH contact is defined in this paper as any SUD or MH outpatient care (e.g., psychotherapy, group or individual therapy, case management, or phone contact)

Metrics data from FY 2012 were linked to the VA Program Evaluation and Resource Center’s FY 2012 *Drug and Alcohol Program Survey* (*DAPS*) and NEPEC’s *MH RRTP Annual Survey* for FY 2012 to create individualized program profile reports. Each report summarized the program’s performance on the *Pre*-*Admission* metrics compared to the national average for similar VA residential SUD treatment programs (i.e., SUD RRTPs or MH RRTPs with a SUD track). See Table 1. The research team then reviewed each report in order to highlight metrics in which the program was a high or low performer compared to the national average and in reference to program-level distributions. This information was used to tailor specific questions in the interview protocol.

### Study population and recruitment

Program managers from the 97 programs were identified using *DAPS* and NEPEC databases and invited to participate in a telephone interview to discuss their program. First, a flyer describing the study was e-mailed to potential participants. If no response was received, a follow-up e-mail was sent a week later which featured select performance data for their program, highlighting both an area of success and an area for improvement (if applicable). A week later, a follow-up telephone call was made to the potential participant to answer any questions about the study. If the point-of-contact could not be reached, a telephone message was left and a subsequent follow-up call was made. To supplement initial recruitment efforts, research staff announced the study on VA-wide SUD representative calls and sent the recruitment flyer to several national VA e-mail groups. At scheduled telephone interviews, participants provided informed consent and were asked permission for interviews to be audiotaped. If the person declined to be audiotaped, then notes were taken. At the end of the interview, participants were asked to recommend front-line staff from their program who might be interested in participating in the study to provide additional perspectives. Interviews were conducted from March 2014 to August 2014. The study protocol was approved by the Stanford University Institutional Review Board.

### Interview content

The interview guide was developed by an interdisciplinary project team with input and feedback from our VA operational partners. It consisted of reviewing a “snapshot” of the program (e.g., occupancy rate), followed by questions about the program’s clinical processes (e.g., screening) and metric performance within the pre-admission, in-treatment, and post-discharge domains. We used a unique data collection technique which combined a semi-structured telephone interview (60–75 min) with Microsoft Lync computer screen-sharing. This method allowed the interviewer to walk the participant, point by point, through the individualized profile report that included their program’s performance relative to the national average for each metric. Participants were asked if the metric data seemed realistic given their knowledge of the program, and what factors may have contributed to their program’s high or low performance on specific metrics. For example, high performers were asked if there is anything they would like to share with other programs on how they achieved success on the metric, whereas low performers were asked if there were barriers and/or resource needs that impacted their performance.

### Qualitative analysis

The interviews were transcribed, cleaned for quality control, and imported into the qualitative data analysis software, ATLAS.ti (Version 7.5.7) [9]. A coding scheme that utilized typical coding techniques for qualitative data [10] was developed to identify common facilitators and barriers described by the participants. The coding scheme included 11 primary codes corresponding to the sections of the interview (e.g., Pre-admission) and two thematic codes (facilitator, barrier). Then, the lead qualitative analyst (LSE) coded each interview according to the 11 primary codes and their section subcodes (e.g., Pre-admission wait time). Next, the lead analyst and two other qualitative analysts (TEP and LM) separately coded quotes in the Pre-admission section based on the thematic codes. The two sets of coded interviews were then merged and queries run separately in ATLAS.ti to extract facilitators and barriers for each pre-admission metric. Lastly, qualitative analysts reviewed the output, *individually* developed lists of barrier themes and facilitator themes, and met to resolve discrepancies and jointly arrive at a consensus of the major facilitator themes, major barrier themes, and their illustrative text components.

## Results

The total number of admissions in FY12 for all programs combined was 14,281 (10,425 admissions to SUD RRTPs; 3856 admissions to MH RRTPs with a SUD track). Among all programs combined, there were 6330 patients with a pre-admission screening (4550 patients in SUD RRTPs; 1780 patients in MH RRTPs with a SUD track). The wait time and engagement while waiting metrics were not calculated if the percentage of patients with a pre-admission screening visit was less than 25%. Roughly a third of the programs (36 out of 97), failed to meet this threshold and, therefore are not included in the summary statistics presented here. In 27 programs, less than 25% of patients had a pre-admission screening visit. For 9 programs, there were no pre-admission screening data.

### Program performance for wait time and engagement while waiting

During FY 2012, the average number of days that a Veteran waited from pre-admission screening to admission into a residential SUD treatment program (“average wait time”) was 17 days for SUD RRTPs (see Table 1), 11 days for MH RRTPs with a SUD track (see Table 1), and 16 days for all programs combined. A total of 60% of Veterans in all programs waited longer than 7 days to be admitted. These Veterans had at least one SUD or mental health contact outpatient encounter during 54% of the weeks while they were waiting (SUD RRTPs: 55%; MH RRTPs with a SUD track: 47%). SUD or mental health contact was defined as psychotherapy, group or individual therapy, case management, or phone contact in a SUD or MH clinic.

### Variation in program performance

Program performance on the pre-admission metrics varied greatly: 0–100% of patients waited longer than 7 days to be admitted, and weeks waiting with at least one SUD or mental health contact (i.e., SUD or mental health outpatient encounter/appointment) ranged from 0 to 100% (0–100% SUD contact only, and 0–94% mental health contact only).

### Interview response rate

A total of 59 interviews were conducted, representing 63 participants (36 female; 27 male) from 44 unique treatment programs (35 SUD RRTPs and 9 MH RRTPs with a SUD track; facility-level response rate of 45%). The interviewees were from 17 of the 21 VA networks and included four joint interviews (i.e., two program staff interviewed together). The majority of providers either had some type of social work degree (40%) or a PhD (35%). MDs and some type of nursing degree each comprised 5%. Program management comprised 37 providers (20 female; 17 male). Similar to the overall providers interviewed, the majority of program management either had some type of social work degree (41%) or a PhD (41%). Four participants declined to be audio-taped but agreed that notes could be taken during the interview. In these cases, an electronic copy of the notes was then uploaded into ATLAS.ti.

### Wait time facilitators

Participants at 11 programs with high performance on either of the two *Wait Time* metrics were asked if they had advice for other programs that may struggle with longer wait times. In response, program staff described process and structural factors that may have contributed to shorter wait times. Several interrelated key facilitators were identified and are described below, with supporting quotations listed in Table 2.

**Table 2:** Interview participants’ perceptions of key facilitators of pre-admission metric performance

Metric	Facilitator	Supporting quotations
I. Wait time	1. Efficient screening processes	“…we don’t schedule face-to-face screens. So there’s no time in between like when we get the consult and then we close the consult, we’re not setting up another additional evaluation meeting with the Veteran that they have to come here which would potentially delay their admission, as far as I know.” “So as we get referrals throughout the day—whether it’s 9:00 or 2 p.m.—we’ll get the referral and we’ll do the screening that day. Prior to the Lean Thinking initiative, we would have one time a day where we would meet at like 11 a.m. But if you get the referral at noon then you gotta wait all the way till 11 a.m. the next day.”
	2. Effective patient flow	“We meet daily for a staffing meeting for 15 minutes to decide, to talk about who’s coming, who’s going, what the plan for the people who are here are, how people are doing in treatment, and that’s when we discuss who’s on the list and how we can get them in.” “…one of the things that we did was add a fourth day and open up the possibility of doing two admissions on 1 day when our census is low. So we’re trying to be a little bit more flexible, a little more accommodating and we’ve also sort of decreased the requirements for being admitted to the program. So we used to have a hard line that they had to have a TB test, for example, prior to admission. Now we can do a test on the day of admission and if they have symptoms, isolate them…”
	3. Available beds	“Okay, we actually had about 120 beds for the Domiciliary which makes us a very large Domiciliary. And that’s a good size for the community and what our needs are. And so generally, it was a fairly short period of time that someone needed to wait to come in.” “And then what’s great about the SA side is that it’s a 45-day program so we can turn around beds a little bit easier.”
II. Engagement while waiting	1. Accessible outpatient services	“…we open all of our groups up to anybody that is interested in participating. We try to individualize that care to the Veteran. So we will let them look at our group schedule and if there’s one or two groups that they can make during the week then we go ahead and invite them in until they get into the inpatient part of the program…And they have some evening groups too that are available like 3 days a week, I believe, 4 days a week, which I think helps.” “There may be a wait to get into a residential bed, but we’ll get you screened quickly and what we do in the interim then if we don’t have a residential bed available, we have an early recovery group, we have individual options for some people and sometimes we’ll even have them do a little bit of our IOP program until a bed opens. So they should be getting services pretty quickly.”
	2. Strong patient outreach	“And we work really hard at calling them if they don’t show up and just really an intensive outreach process. I think because it seems the nature of the population is that they easily disappear, so we work really hard to try not to let that happen.”
	3. Strong encouragement of pre-admission outpatient treatment	“So, anyone on the list, we tell them if you’re coming to outpatient, you’re staying involved and we can see that you’re maintaining and motivated and someone doesn’t show, well, we’re going to come to outpatient and say, ‘Hey somebody didn’t show’ or ‘Someone left AMA.’ And so, it gives you a better chance of getting into the program quicker and the fact that we’re all on the same floor, I think that, you know, they can check in with us on a daily basis even if they want to.” “Well, I think that one of the things is that we tell people it’s an expectation. So, when we screen them, and say our wait is like two and a half weeks at this point or something, we tell them a couple of things, that we’re really requiring you to do outpatient unless there’s really some legitimate reason, like you live too far away or whatever, to not come.” “But when the patients are screened, they automatically are enrolled or scheduled for, number one, a pre-treatment group that we call “Preparation Group,” that meets twice a week and that’s done in the outpatient clinic.”

#### Wait time facilitator 1: efficient screening processes

Many participants described ways in which they optimized efficiency in their screening and assessment processes, which in turn may have contributed to shorter wait times for Veterans waiting to enter the program. Among the strategies employed by programs were (a) keeping the number of assessments or screening appointments per patient to a minimum, and (b) flexibility in scheduling screening appointments or other processes to accommodate more timely screenings.

#### Wait time facilitator 2: effective patient flow

The residential treatment program process includes waiting for admission, going through the process of being admitted, working through the components of the program, and the discharge process. In some cases, these processes overlap. Effectively managing patient flow, whether at an individual, team, or facility level, appeared to be integral in efforts at keeping program wait times below the national average. Key facilitators included: (a) regularly-scheduled meetings to review patient status to make sure they are not unnecessarily stalled between processes and (b) flexible admissions processes and requirements.

#### Wait time facilitator 3: available beds

The availability of beds was another factor that was commonly attributed to lower wait times. How long a Veteran waits to enter the program may be reflected, in part by the sheer number of beds that a residential treatment program has available at any given time. Several participants described how their program’s high capacity may have positively influenced their program’s performance on the wait time metrics.

### Wait time barriers

Participants at 14 programs with low performance on either of the two *Wait Time* metrics reported the following reasons and challenges. See Table 3.

**Table 3:** Interview participants’ perceptions of key barriers affecting pre-admission metric performance

Metric	Barrier	Supporting quotations
I. Wait time	1. Lack of beds	“Well, we only have 20 beds and we’re serving four hospitals…We have 20 beds for a lot of people.” “…there were two medical centers in the X area up until 2011…Basically the substance abuse program was consolidated here at the X Hospital in X. So prior to that, the SUD program was actually out at a separate VA medical center and we had more beds there and little to no waitlist. So ever since the move here and the consolidation, we actually lost a significant number of beds and ever since then basically wait time has been an ongoing issue.”
	2. Poor staffing levels	“…we had only one psychiatrist who was doing the work so we had to keep our census at half…” “We were pretty well staffed and then we had a kind of an exodus of social workers and all at once. Then we had to rehire, and that took a long time. And then at that same time, we just kept getting more and more referrals, more and more applications.”
	3. Length of stay	“The other side of it could be that our length of stay is too long. We are working on that and have revamped our program to have an eight-week option, as well as a longer option. So, we’re trying to address that part of it.” “In addition to a variable length of stay for folks, I mean, one of the things that we struggled with was if people needed more time than we’re able to extend them; if we extend them, it creates a longer wait for people on that admissions list.”
II. Engagement while waiting	1. Poor staffing levels	“I think even adding the X CBOCs, there’s 12 of them up north; only this month have they gotten CBT SUD groups in all the rural areas. So, you can imagine our continuity of care fallouts because they were sending guys out, you know, to the boondocks with no SUD providers available even by CBT.” “A lot of our people would be seeing people in CBOCs, and I would very much doubt if those people are stop coding, you know, for SUD. We have a single social worker or whatever trying to cope. And then in this medical center, our SUD is way understaffed. So, they don’t give a lot of outpatient contact to anybody. So, it’s very hard to get in.” “We’ve talked about having a waiting for treatment group. But that has been something that staffing-wise we have not been able to pull off.”
	2. Socioeconomic barriers	“Well, right now, out in one of the X CBOCs, the bus station is actually like five or six miles away, the closest bus stop to the CBOC. So, you couldn’t even take a bus to get to the CBOCs…” “I think for a lot of these people who are more indigent, or they don’t have cars. Or, if they even have cars, they might not have what they call gas money to get to the place. I think they tend to no-show a lot, or they don’t’ have a way of getting in to treatment.”
	3. Low patient motivation	“Sometimes it’s motivation as well. I mean sometimes they’re using hard, they’re drinking hard or whatever. They’re just not that motivated to get up and come into a weekly group. They’ll show up for their admission appointment because at that point they’re like ‘Okay I’m ready to dry out and get serious.’” But up until that point, they’re continuing to party and use.” “We don’t have a group, because we tried to do a group for people waiting and nobody showed up. So we do offer individual visits and rarely—I’d say less than 10% of the people waiting take advantage of that.”

#### Wait time barrier 1: lack of beds

Many participants indicated that the lack of available beds impacted how long Veterans waited to enter their program in FY 2012. Key factors affecting bed availability were high demand and reduction of bed availability.

#### Wait time barrier 2: poor staffing levels

Participants indicated that staff shortages (psychiatrists, social workers) at the residential programs hindered their ability to admit patients more quickly and having fewer staff at the smaller Community Based Outpatient Clinics (CBOCs) delayed important processes such as medical clearances.

#### Wait time barrier 3: length of stay

Several participants acknowledged that program length or extending a Veteran’s length of stay after program completion when warranted can unintentionally lead to some Veterans waiting longer to enter the program.

The programs in which we interviewed program management and front-line staff were more likely to be low performers on the 7-day wait metric. However, among these programs in which patients waited longer than 7 days to be admitted, they were more likely to be high performers with respect to the proportion of waiting weeks with at least one SUD/mental health outpatient encounter (these data exclude programs with less than 25% pre-admission screening, for which these metrics were not calculated).

### Engagement while waiting facilitators

Participants at 17 programs with high performance on any of the three *Engagement While Waiting* metrics were asked if there were particular aspects of their pre-admission processes that may have facilitated the provision of outpatient SUD and/or MH treatment while Veterans were waiting to enter the program. Several interrelated key factors were identified and are described below, with supporting quotations listed in Table 2.

#### Engagement while waiting facilitator 1: accessible outpatient services

Programs that were successful at engaging Veterans pre-admission leveraged their ability to offer and connect Veterans to SUD and mental health outpatient services within and/or outside the VA. In addition, some participants emphasized to Veterans the importance of staying connected to their outpatient providers if they were already accessing VA outpatient services. Fourteen of the 17 programs with high performance on this metric tried to engage Veterans waiting for admission in treatment groups such as pre-existing groups within the VA residential treatment program and VA intensive outpatient programs. Eight of the 17 high performing sites offered Veterans the opportunity to attend an established pre-admission group.

#### Engagement while waiting facilitator 2: strong patient outreach

In general, participants described a variety of motivational and outreach strategies believed to be instrumental in successfully engaging Veterans in treatment activities while waiting for admission. Program staff commonly reached out to and followed up with Veterans by telephone (e.g., daily, weekly, biweekly).

#### Engagement while waiting facilitator 3: strong encouragement of pre-admission outpatient treatment

Strongly encouraging pre-admission group participation was perceived to have a favorable impact on the degree to which Veterans engaged in treatment activities prior to admission. One participant illustrated how the staff incentivized Veterans to attend outpatient treatment by letting them know that pre-admission group participation would “improve their chance of getting into the program quicker” (Table 2).

### Engagement while waiting barriers

Participants at 14 programs with low performance on any of the three *Engagement While Waiting* metrics, relative to the national average for their program type, were asked about barriers they faced in facilitating pre-admission engagement. The most commonly-cited barriers are described below, with corresponding supporting quotations listed in Table 3.

#### Engagement while waiting barrier 1: poor staffing levels

One of the primary obstacles voiced by participants was staffing constraints, which can make it difficult for program staff to provide optimal access to outpatient treatment.

#### Engagement while waiting barrier 2: socioeconomic barriers

Participants also noted that due to socioeconomic factors, Veterans themselves find it difficult to access outpatient treatment even when it is available. The most common socioeconomic barriers voiced by participants were (a) geographic location/driving distance to outpatient treatment, (b) lack of public transportation, and (c) financial constraints, such as lacking money to buy gas.

#### Engagement while waiting barrier 3: low patient motivation

Fewer participants perceived that Veterans themselves may hinder their own opportunities to engage in treatment. In other words, some Veterans do not avail themselves of outpatient services.

## Discussion

This study aimed to: (1) Develop metrics for two pre-admission processes: *Wait Time* and *Engagement While Waiting*, and (2) Interview program management and staff about program structures and processes that may contribute to performance on these metrics. The first aim sought to close a gap in the VA’s existing performance metrics in order to identify high-value targets for quality improvement. The second aim recognized that not all key processes are reflected in the administrative data, and even when they are, new insight may be gained from viewing these data in the context of day-to-day clinical practice. We hypothesized that speaking directly to residential program staff may reveal innovative practices, areas for improvement, and factors that may explain system-wide variability in performance.

In this project, we learned that program-level performance on the pre-admission metrics was highly variable. Across programs, 0–100% of Veterans waited longer than 7 days to be admitted (average: 60%). For these Veterans, at least one SUD or mental health contact occurred in 3–100% of the waiting weeks (average: 54%). This degree of clinical variability is consistent with previous studies and evaluative reports of SUD treatment in VA SUD RRTPs [11, 12].

We found that program management and staff shared common perceptions of the structures and processes that may have contributed to their program’s high or low performance on the pre-admission metrics. This information can be useful for designing interventions to improve access to residential SUD treatment and reduce system-wide variability. Efficient screening processes and effective patient flow are processes that theoretically could be implemented across programs to help reduce wait times if there is good communication among all levels of staff. The availability of beds which emerged as a key determinant (barrier and facilitator) of wait times is structural, more static and largely dependent upon factors external to the program (e.g., facility/VA budget).

Given resource constraints, it may be tempting to concentrate efforts on other processes such as efficient screening and effective patient flow to reduce wait times, rather than increasing number of beds. However, systems can only become so efficient and resources spent on efficiency may take away resources from more beds. In addition, effective patient flow often depends upon sufficient staffing within the program itself and its ancillary components, which can be costly depending on the program’s needs. When staffing levels are suboptimal, this can potentially impact how quickly Veterans can be admitted to and discharged from the program. Most of the interview comments related to staffing levels were general in nature, although some respondents mentioned specific staffing needs. As noted in Table 3, one respondent indicated that with only one psychiatrist doing the work, they were compelled to reduce their census by half. Another program lacked sufficient social workers, particularly during a time of increased referrals and applications, which staff perceived as potentially affecting wait time. Therefore, it is important to advocate for more beds and sufficient staffing along with low-cost, effective processes that may also ease wait times. It is also worth noting that although programs in which we interviewed staff were more likely to be low performers on the 7-day wait metric, among the low performers, they were more likely to be high performers with respect to the proportion of waiting weeks with at least one SUD/mental health outpatient encounter. This finding is heartening in that it appears that programs are seeking to provide outpatient care during this critical window of time despite difficult circumstances.

We found that successfully engaging Veterans in outpatient treatment while they wait appears to depend heavily on efforts by staff, at the individual provider, program, and facility level. Overall, good internal and inter-facility communication appears vital to providing Veterans with timely access to residential treatment, and may also have positive ripple effects (e.g., engaging Veterans pre-admission may lead to fewer “no shows” to group treatment activities).

Poor staffing levels and socioeconomic barriers (e.g., lack of transportation) which were often perceived to be beyond the staff’s control may be difficult to surmount as they are dependent on sufficient financial resources at the medical center. Yet, they are important to address as they potentially affect quality of care. As noted in Table 3, one respondent indicated that there had been a shortage of SUD providers in the Community Based Outpatient Clinics (CBOCs) “up north” and “no SUD providers available even by CBT” in rural areas, which impacted the type of outpatient care available to Veterans waiting for admission to the program. Another respondent mentioned that their program has only one social worker and that their medical facility is “way understaffed” in terms of SUD providers. Staffing costs will invariably depend on the type of staff required per site as well as the particular needs of the program.

Some programs however, showed resiliency and creativity in the face of these challenges. For instance, several programs were in the midst of advocating for transportation “workarounds” or creating their own (e.g., asking other Veterans if they were willing to give others a ride). Another program had not yet set up a group for Veterans who are waiting, but found peer support specialists to be particularly helpful in the interim. Others cited strategies (e.g., telemental health for patients in rural areas) that their program would benefit from having.

While some patients who do not engage in outpatient treatment may truly lack motivation, it is possible that some factors perceived as low motivation (e.g., failing to show up for a group or individual session while waiting), may stem from things beyond their control. Veterans struggling with issues such as transportation/housing may not be able to avail themselves of outpatient care, and for some, these factors may have been the driving force for seeking residential care in the first place. As such, these Veterans would also be precluded from an improved chance of getting into a residential program if they are incentivized to attend outpatient treatment—a strategy previously indicated by one provider as facilitating engagement while waiting. Any unintended consequences like this deserve attention to ensure equitability in access to services.

The potential value of these pre-admission metrics is significant given that they provide VA leadership, as well as program management and staff, with a way to easily monitor the access, quality, and efficiency of residential SUD treatment programs. In response to an identified need to rapidly develop metrics for monitoring access to residential treatment, MHS and OMHO built upon the initial metrics developed by this study and implemented wait time metrics for the residential programs in October of 2014. These metrics continue to be refined with quarterly updates provided to the field and are part of a broader set of metrics that have been developed to understand access to residential treatment. By operationalizing this wait time metric, residential SUD treatment staff can view their program’s performance over time and identify opportunities for implementation and quality improvement efforts. It has been a critical component to ensuring a clear understanding of residential access and guiding strategic planning efforts at the regional and local level to improve access. Further, MHS and OMHO are working towards refinement and implementation of the remaining metrics. The initial data provided by this study serves as baseline data and a point of comparison as new metrics are implemented.

The identification of common perceived facilitators and barriers offer opportunities for clinical leadership and health services researchers to craft interventions to improve timely access to services and disseminate “best practices” to the field. Although some of the identified “best practices” may appear to simply be the expected standard of care, the responses from interview participants reflect how a Veteran’s quality of care is critically tied to the consistent execution of these tasks. In addition, the support, cooperation, and communication among program staff and facility-wide appeared to play a vital role.

We recognize some limitations of this study. We did not interview peripheral VA staff members (e.g., outpatient personnel who conduct pre-admissions screenings) that are located at another site/campus within the facility, nor did we interview Veterans. In addition, less than half of the VA’s residential SUD programs were represented in the interviews, so our findings may not be generalizable to all VA residential SUD programs or to residential SUD programs outside the VA system. Moreover, there was substantially less representation from MH RRTPs with a SUD track (20%), compared to SUD RRTPs (80%). While we believe that provider interviews can be a beneficial component of quality improvement, we did not evaluate the effect of conveying what we learned back to our stakeholders. The next step would be to assess the potential value of interviewing program management and front-line staff while developing performance metrics. Since this endeavor can be labor-intensive, this reaffirms the need for evaluation. Despite these limitations, this study successfully created new metrics to monitor performance and identify areas for improvement, as well as an inventory of potential solutions informed by residential program management and front-line staff.

## Conclusion

Pre-admission process metrics developed in this study have been refined and are proving to be helpful for monitoring residential SUD treatment programs within the VA. Interviewing program management and staff about drivers of performance metrics can play a critical and complementary role by identifying innovative and other strong practices, as well as high-value targets for quality improvement. Key facilitators of pre-admission processes in high-performing facilities may offer programs with lower performance useful strategies to improve the quality of their pre-admission processes.

## Abbreviations

VA: United States Department of Veterans Affairs
SUD: substance use disorder
SUD RRTPs: SUD Residential Rehabilitation Treatment Programs
MH RRTPs: Mental Health Residential Rehabilitation Treatment Programs
FY: fiscal year
MHIS: Mental Health Information System
NEPEC: Northeast Program Evaluation Center
MHS: VA Mental Health Services
OMHO: VA Office of Mental Health Operations
OIG: Office of the Inspector General
DAPS: VA Drug and Alcohol Program Survey
CBOCs: Community Based Outpatient Clinics
